# Analysis of microbial contamination during use and reprocessing of surgical instruments and sterile packaging systems

**DOI:** 10.1371/journal.pone.0280595

**Published:** 2023-01-20

**Authors:** Lena Dreikausen, Bernd Blender, Milena Trifunovic-Koenig, Florian Salm, Stefan Bushuven, Bianka Gerber, Matthias Henke

**Affiliations:** 1 Front End Innovation & Materials (FEIM), Aesculap AG, Tuttlingen, Germany; 2 Institute for Infection Control and Infection Prevention, Health Care Association District of Constance, Konstanz, Germany; 3 Institute for Anesthesiology, Intensive Care, Emergency Medicine and Pain Therapy, Hegau Bodensee Hospital Singen, Singen, Germany; VIT University, INDIA

## Abstract

A surgical site infection (SSI) is one of the most common surgical complications. This study analyzed different sources of microorganisms in the air, on reusable surgical instruments, and the outer surface of sterile packaging systems during the use and reprocessing of sterile goods (from the operating room (OR) to the Central Sterile Supply Department (CSSD)). The microbial load in the air was analyzed via active air sampling and settle plates. Furthermore, the airborne particle load was measured by a particle counter. Contact agar plates were used to determine the microbial load on surgical instruments and sterile packaging systems. The highest average microbial and particle load was measured in the air of the OR (active air sampling: max. 56 CFU/m^3^; settle plates: max. 9 CFU; ≥0.3 μm particles in size: 1,958,403 no./m^3^). However, no microbial load (0 CFU) was detected on surgical instruments sampled in the OR. The outer surface of stored sterile packaging systems showed a maximal microbial load of 64 CFU. The most common identified pathogen was coagulase-negative staphylococci. Compared to properly reprocessed reusable surgical instruments and sterile packaging systems, the air still seems to be the primary potential source of microbial contamination, especially within the OR.

## Introduction

Surgical site infections (SSI) are one of the most common complications after surgery. These infections, affect the superficial or deep tissues of the operating site [1]. SSIs are associated with a longer postoperative hospital stay, additional surgical procedures, intensive care, increased morbidity and mortality, and higher costs [2]. The annual epidemiological report of the European Centre for Disease Prevention and Control (ECDC) revealed 10,149 SSIs in 2017 and demonstrated that the percentage of SSIs procedures varied from 0.5% to 10.1%, depending on the type of procedure. The highest percentage was detected for open colon surgery with 10.1%, followed by other surgeries, e.g., 1.0% for hip prosthesis surgery and 0.5% for knee prosthesis surgery. The most common reported bacteria causing SSI is *Staphylococcus aureus* (21.5%), followed by *Escherichia coli* (13.9%), *Enterococcus* species (11.9%), and coagulase-negative staphylococci (11.0%) [3].

Various preoperative and postoperative measures can be taken to prevent SSIs, such as nasal decolonization, preoperative showers, hair removal, antibiotic prophylaxis, and surgical hand preparation [1, 4, 5]. Reprocessing of reusable surgical instruments depicts one of the multimodal exogenous sources of bacteria causing SSIs [6]. For reusability, these instruments are cleaned and thermally disinfected within the Central Sterile Supply Departments (CSSD). Afterward, they are inspected, packed into trays and sterile packaging systems, sterilized, and stored until reused in the operating rooms (OR) [1]. These instruments could be contaminated by microorganisms at different steps during use and reprocessing. For example, intraoperatively during surgical usage [7–10] or after their handling within the CSSD [10–12]. Furthermore, opened sterile operating-room trays containing sterile surgical instruments could be contaminated while they are left open in the protective area of the OR [13, 14].

In this context, the air within the OR is one common exogenous source of microbial contamination. Microorganisms, such as bacteria, viruses, or fungal spores, exist everywhere in the air as bioaerosols. The particle size of bioaerosols varies between 0.3–100 μm in diameter (microbial cells ca. 1 μm, virus particles 1 nm, fungal spores >1 μm). Microbial cells and virus particles are often attached to skin cells, dust, and other organic or inorganic material [15–17]. Different conventional ventilation and conditioning systems with a mixed or turbulent flow are used for all types of surgeries to reduce the bioaerosol- and particle load in the OR and to guide their flow direction. Laminar airflow (LAF) systems are designed to redirect particle-free air, also called ultra-clean air, over the aseptic operating field at a uniform velocity, leading particles and bioaerosols away. Additionally, most operating rooms are maintained at positive pressure compared to corridors and other surrounding areas to prevent airflow from less clean areas into the OR [1, 5]. The correlation between air ventilation systems within the OR and postoperative infections was described very early [18–20]. However, these and other studies demonstrate that despite the ventilation system, the particle and microbial load within the OR is never fully eliminated, even in an empty OR.

The aim of this explorative study is to identify the degree of exogenous microbial contamination risk of reusable surgical instruments during usage within the OR and various steps of reprocessing within the CSSD. Accordingly, the microbial load on used and unused instruments, on the outer surface of sterile packaging systems, and in the air was measured during use and reprocessing of sterile goods (before surgery, during surgery, after surgery, disposal, after cleaning & disinfecting, after inspection & packaging, after sterilization, storage, and transport).

## Materials and methods

### Hospitals and analyzed rooms

For this explorative study, seven hospitals in Germany were contacted. Three were included due to their proximity to the external laboratory for logistical ease and high quality-assuring transport conditions of the samples. A total of nine surgical procedures were monitored, including neurosurgery, orthopedic surgery, and vascular surgery. The surgical wound classification was classified as “clean” in these surgeries. Each of the nine surgeries was the first surgery of the day (early in the morning). In each hospital three different surgeries were analyzed on three different days. Thereafter, the other rooms (CSSD, sterile storage area, etc.) were monitored. Each OR was equipped with a fully air-conditioned system with a laminar airflow and a high-efficiency particulate air (HEPA) filter, H13 or H14. The HEPA filters remove particles corresponding to DIN EN 1822–1:2019 with an efficiency of 99.95% (H13) or 99.995% (H14). The ORs were classified in reference to DIN 1946–4 as class 1a rooms (3 filtration steps). Furthermore, these ORs were under positive pressure in relation to the adjacent rooms. The CSSD was classified as class 1a or class 2 rooms. The class 2 rooms were equipped with a two-filtration-step system (without HEPA filter) and were fully air-conditioned.

### Timing and locations of sampling

**Fig 1:**
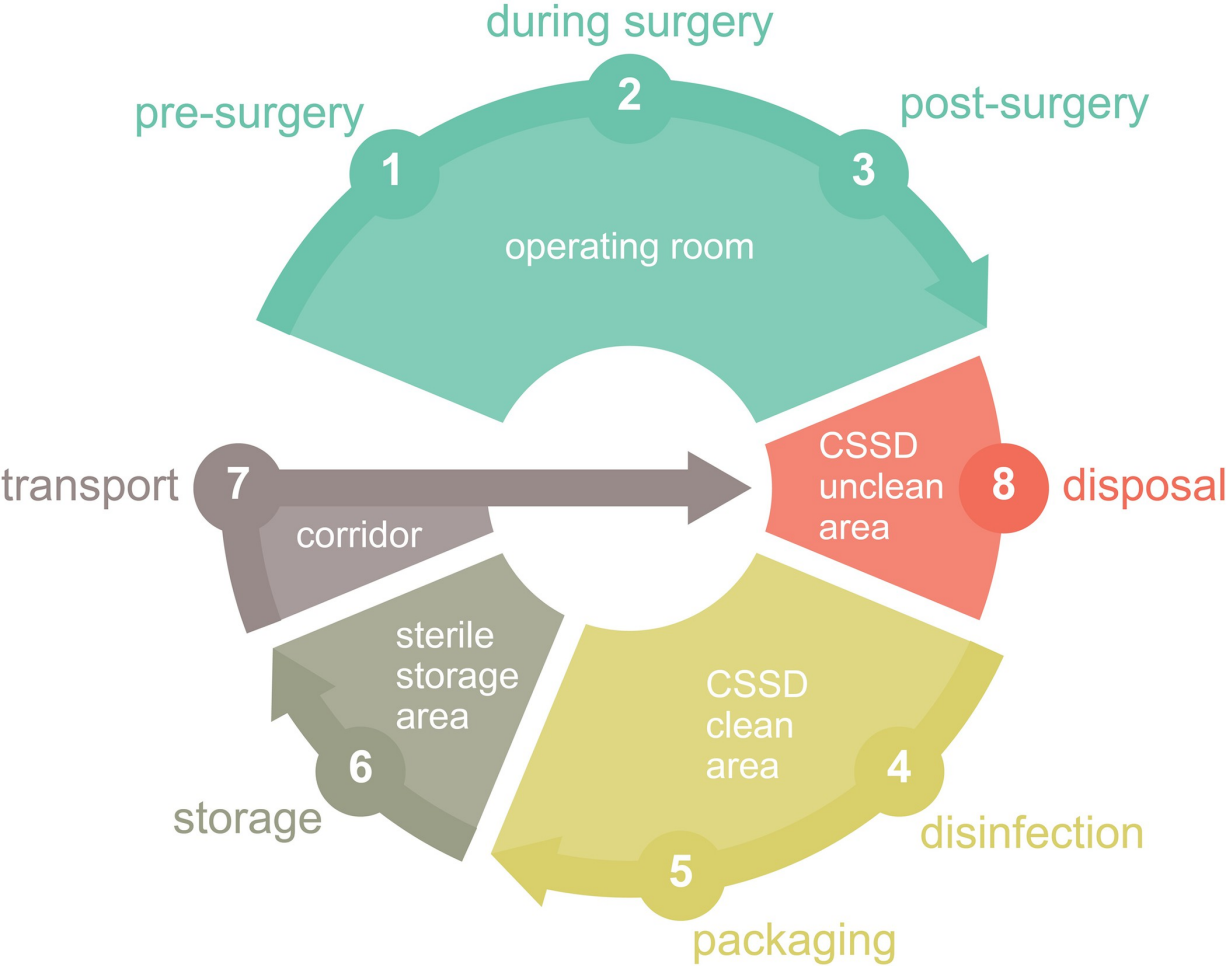
Steps and locations of sampling during use and reprocessing of sterile goods. The microbial load on instruments was sampled pre-surgery (1), post-surgery (3), after disinfection (4), after packaging (5), storage (6), and disposal (8). Samples on the outer surface of sterile packaging systems (reusable containers) were sampled pre-surgery (1), after disinfection (4), after packaging (5), storage (6), and disposal (8). Airborne microbial load (via impactor and settle plates) and airborne particle load (using particle counter) were measured at all steps and locations of sampling during use and reprocessing (1–8).

All samples were taken by qualified personnel during use and reprocessing of sterile goods in the order depicted in Fig 1. The microbial load on the instruments, on the outer surface of sterile packaging systems, and the microbial and particle load in the air was measured during all steps of use and reprocessing.

The first samples were taken before the first surgery of the day in the empty OR (“pre-surgery (1)”) (Fig 1). Thereafter, samples were taken intraoperatively (“during surgery (2)”) and immediately after the first surgery of the day within the same operating room (“post-surgery (3)”) (Fig 1). The next step is usually the disposal in the unclean area of the CSSD. However, to avoid contamination due to the transfer of the measuring devices, the order of sampling was changed, and samples were taken last in the unclean area of the CSSD. Thus, after the measurements in the OR, the next samples were taken in the clean area of the CSSD, directly after the reusable surgical instruments had been cleaned, thermally disinfected in a washer-disinfector (regularly validated according to ISO 15883), and dried (sampling description: “disinfection (4)”) (Fig 1). The subsequent samples were taken after the instruments were inspected and packed into instrument trays by CSSD staff without gloves, but with sanitized hands (“packaging (5)”) (Fig 1). At this step of reprocessing, the air sampler and particle counter were placed on the table where surgical instruments were packed. No samples were collected directly after steam sterilization (134°C, 3 bar, 3–5 min) as these sterile products were transported directly to the sterile storage area. Therefore, the next samples were taken within the sterile storage area (“storage (6)”). Subsequently, samples were collected on the transport ways from the sterile storage area to the OR in corridors or elevators (“transport (7)”) and finally in the unclean area of the CSSD (“disposal (8)”).

### Determining the microbial load on surgical instruments and sterile packaging systems

Surgical instruments were sampled in an order as described above (Fig 1) during use and reprocessing wearing sterile surgical gloves. At the different steps and locations of sampling (Fig 1) (pre-surgery, during surgery, post-surgery, disinfection, packaging, storage, transport, and disposal), instruments were randomly sampled from different instrument trays. CASO-contact (tryptic soy agar) agar plates (Ø 55 mm) (Merck Chemicals GmbH, Darmstadt, Germany) were pressed onto the part of the instrument which was in contact with the patient during usage. After contact with the agar plates, all sampled surgical instruments were cleaned, disinfected, and sterilized before reuse. A total of 245 reusable surgical instruments from nine surgeries were analyzed throughout the whole study.

To determine the microbial load on sterile packaging systems during use and reprocessing (1–8), contact agar plates were used and pressed on the outer surface of reusable containers (definition of a reusable container according to ISO 11607–1). After contact with agar plates, the reusable containers were cleaned, disinfected, packed with instrument trays and surgical instruments, and sterilized. Reusable containers of three different manufacturers with different models, ages, and at different conditions were analyzed. The choice of sampling reusable containers was based on easier handling during sampling in a non-sterile environment.

### Measuring the microbial load in the air via active air sampling

We used a SAS SUPER 100 impactor (VWR International GmbH, Bruchsal, Germany) to determine the total airborne microbial load during the use and reprocessing steps (Fig 1). The impactor air sampler was equipped with a tryptic soy agar plate (Ø 90 mm) (Merck Chemicals GmbH, Darmstadt, Germany). The air sampler was placed on a separate table (height 1 m) with a minimum of 1 m away from walls and other items at every sampling location (1–8). Measurements within the OR (1–3) were performed outside the sterile field.

First, the flow rate of 100 L/min for 5 min was selected. Then, the results were multiplied by factor 2 (total volume of 1000 L) to express the results as Colony Forming Units per m^3^ (CFU/m^3^). The impactor was cleaned with disinfection wipes before each room change and manually reactivated at each new sampling location to avoid contamination. A total of 107 air samples were taken. Air sampling was performed once at every step of use and reprocessing, except during surgery (2) when measurements were taken every 15 min.

### Passive air sampling via settle plates

Passive air sampling was performed in all three hospitals for all 9 surgeries using settle plates. CASO-contact (tryptic soy agar) agar plates (Ø 55 mm) (Merck Chemicals GmbH, Darmstadt, Germany) were left open for a defined period (10 min). The agar plates were placed near the air sampler (air sampler turned off, 1 m away from the floor, and 1 m away from walls and other items). A total of 100 samples were taken via settle plates throughout the whole study.

### Microbial quantification and identification

All agar plates were sent to an external laboratory and incubated for 48 hours at 36°C +/- 1°C for bacterial growth, and five days at room temperature for fungal growth, before measuring the total bacterial or fungal count. Microbiological results are expressed as colony forming units (CFU). Microorganisms were identified by optical identification or by matrix-assisted laser desorption time-of-flight mass spectrometry (MALDI TOF).

### Analyzing the airborne particle load

Airborne particles were counted by the Lasair III Aerosol Particle Counter (Particle Measuring Systems, Boulder, CO, USA). This light-scattering particle counter counts particles of different sizes of (Ø 0.3, 0.5, 1.0, 5.0, 10.0, 25.0 μm in diameter) at a flow rate of 28.3 L/min. The particle counter was placed near the SAS impactor at each sampling location (Fig 1). Particle concentration was expressed as the number of particles per m^3^ (no./m^3^).

### Statistical analysis

The independent-samples Kruskal-Wallis Test was performed to determine whether the computed differences amongst the hospitals were statistically significant. The Wilcoxon Sign-Rank Test was used to compare the microbial load on the outer surface and instruments sampled from the same reusable container in the sterile storage area (storage (6)). To examine the differences in the airborne microbial load pre-, during, and post-surgery (1–3), we used the non-parametric related samples Friedman’s two-way analysis of variance by ranks test. The level of statistical significance in all analyses was set at α = 0.05. The statistical analysis was performed using SPSS Software Version 27 (IBM SPSS Statistics for Windows, Version 27.0. Armonk, IBM, New York, USA). The diagrams and the statistical analysis within the diagrams were created with Statistica Version 14.0.0.15 (Tibco, Palo Alto, USA).

## Results

### Microbial load on instruments and the outer surface of reusable containers

Microbial contamination sampled using contact agar plates was found on surgical instruments taken from the CSSD area prior to sterilization (Table 1) (sampling steps 4,5,8). Whereas no microbial load (0 CFU) was detected on surgical instruments sampled pre-, during, and post-surgery (1–3). At this step of sampling, no difference was observed whether the instruments were used during the surgery (2) and were in contact with blood or left open on the sterile table and left unused (3). Furthermore, no microbial load (0 CFU) was detected on sterile instruments taken from reusable containers which were stored in the sterile storage area (6). The highest microbial load of 59 CFU on instruments was sampled in the unclean area of the CSSD prior to manual cleaning (disposal (8)). Furthermore, no significant difference was found between the hospitals for disinfection (4), packaging (5), and disposal (8) (Kruskal-Wallis test p >0.05).

**Table 1:** Microbial load measured on instruments and the outer surface of reusable containers.

		(1)	(2)	(3)	(4)	(5)	(6)	(7)	(8)
		Pre-surgery	During surgery	Post-surgery	Disinfection	Packaging	Storage	Transport	Disposal
**On instruments**	**Mean ± SD**	0 ± 0	n.a.	0 ± 0*	0 ± 1	2 ± 4	0 ± 0	n.a.	5 ± 14
	**Min—Max**	0–0	n.a.	0–0	0–3	0–20	0–0	n.a.	0–59
**Outer surface of containers**	**Mean ± SD**	4 ± 9	n.a.	n.a.	11 ± 3	7 ± 8	18 ± 19	n.a.	3 ± 3
	**Min—Max**	0–20	n.a.	n.a.	9–13	1–12	0–64	n.a.	0–6

*used and unused instruments

Depicted are the mean values, standard deviations (SD), minimum (Min), and maximum (Max) values of the microbial load measured on instruments and on the outer surface of reusable containers during use and reprocessing (1–8). Microbial load is shown as colony forming units [CFU], (n.a. = not applicable).

Among the 245 sampled instruments, 27 (11%) contained bacteria or fungi. A total microbial load of 228 CFU could be isolated from instruments, of which 55 (24%) were SSI-relevant. Coagulase-negative staphylococci was the most common pathogen (32/228–14%).

A total of 40 samples were taken from the outer surface of reusable containers. Of these, 34 (85%) contained bacteria or fungi. The highest microbial load of 64 CFU was detected on the outer surface of reusable containers taken from the sterile storage area (storage (6)). Among the bacteria isolated on the outer surface of reusable containers 47% were coagulase-negative staphylococci. There was a statistically significant difference with a strong effect size between the outer surface of the reusable containers and the instruments contained in the same reusable containers (Wilcoxon Sign-Rank Test: Z = 2.67; p = 0.008; r = 0.85).

### Airborne microbial load

**Fig 2:**
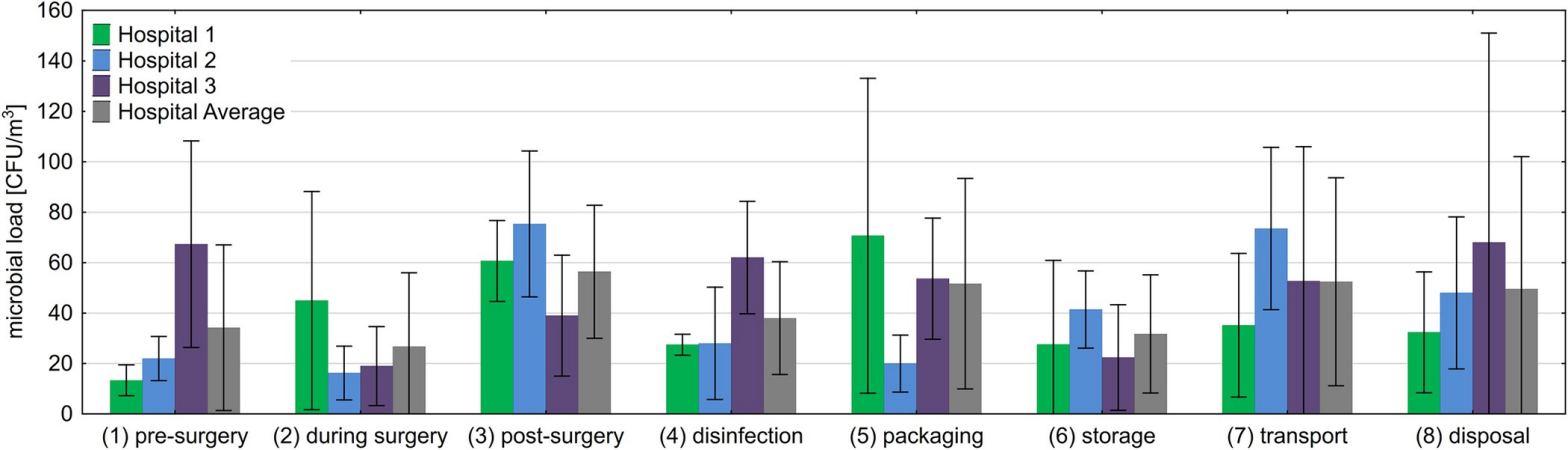
Airborne microbial load measured via active air sampling during use and reprocessing. Mean values and standard deviations of the airborne microbial load of the three hospitals (hospital 1–3, colored bars) and the average load of all hospitals (grey bars) measured during use and reprocessing of sterile goods (1–8) are shown as colony forming units/m^3^ [CFU/m^3^].

Two methods were used to determine the microbial load in the air: active air sampling via an impactor and passive air sampling via settle plates. The average microbial load of active air sampling was highest post-surgery (3) with 56 CFU/m^3^ and lowest during surgery (2) with 27 CFU/m^3^ (Fig 2). However, the Friedman test indicated that the highlighted differences were not statistically significant (χ^2^(7) = 3.56, *p* = 0.17) for the first sample taken pre-, during, and post-surgery (1–3). In addition, neither the mean nor maximal microbial loads measured at different steps of reprocessing differed across the hospitals (Kruskal-Wallis test for all reprocessing steps (1–8) p > 0.05).

Of these 107 samples, 105 (98%) contained bacteria and fungi isolated by active air sampling. A total amount of 4,327 CFU was isolated. Of these microorganisms, 1,225 (28%) were SSI-relevant, and among these, coagulase-negative staphylococci were the most common pathogen at 1,223 (28%).

A total of 100 samples were collected via passive air sampling, of which 41 (41%) contained microorganisms (Table 2). A total amount of 91 CFU could be isolated, of which 44 (48%) were SSI relevant. All SSI-relevant bacteria were coagulase-negative staphylococci. The highest microbial load of 9 CFU was detected during active surgery (2). In the other steps of sampling (e.g., within the CSSD (4,5,8), storage (6), or transport (7)), the microbial load detected on settle plates was very low.

**Table 2:** Analysis of the airborne microbial load during use and reprocessing using settle plates.

		(1)	(2)	(3)	(4)	(5)	(6)	(7)	(8)
		Pre-surgery	During surgery	Post-surgery	Disinfection	Packaging	Storage	Transport	Disposal
**Hospital 1**	**Mean ± SD**	0 ± 0	1 ± 1	1 ± 1	0 ± 0	1 ± 1	1 ± 1	2 ± 1	0 ± 0
	**Min—Max**	0–1	0–3	0–2	0–1	0–1	0–1	0–3	0–0
**Hospital 2**	**Mean ± SD**	0 ± 0	0 ± 0	3 ± 1	2 ± 1	0 ± 0	0 ± 0	1 ± 1	1 ± 1
	**Min—Max**	0–0	0–1	1–4	0–1	0–0	0–1	0–2	0–1
**Hospital 3**	**Mean ± SD**	2 ± 2	1 ± 3	3 ± 2	2 ± 1	0 ± 0	0 ± 1	0 ± 0	3 ± 2
	**Min—Max**	0–4	0–9	1–5	1–4	0–0	0–1	0–0	0–4
**Average**	**Mean ± SD**	1 ± 1	1 ± 0	2 ± 1	1 ± 1	0 ± 0	0 ± 0	1 ± 1	1 ± 1
	**Min—Max**	0–4	0–9	0–5	0–4	0–1	0–1	0–3	0–4

Mean values, standard deviations (SD), minimum (Min) and maximum (Max) values of the airborne microbial load, measured in all three hospitals during use and reprocessing (1–8), are depicted as colony forming units [CFU].

### Airborne particle load

Microbes, such as bacteria and fungi, are often linked to particles as so-called bioaerosols. Therefore, the airborne particle load was measured with a particle counter on five of the nine different days (Table 3). The highest recorded particle load of 0.3 μm particles was 1,958,403 no./m^3^ during active surgery (2). Within the packaging area in the CSSD (5), the highest particle load of 0.5 μm particles was 639,528 no./m^3^ and of 1 μm particles was 458,094 no./m^3^. In the area after cleaning and disinfection (4) within the CSSD, a maximum amount of 5 μm particles of 12,956 no./m^3^ and 10 μm particles of 1,818 no./m^3^ was measured. Directly after surgery (post-surgery (3)) the highest particle load of 25 μm particles 233 no./m^3^ could be recorded.

**Table 3:** Airborne particle load measured during use and reprocessing (1–8) using a particle counter.

Particle size in diameter		(1)	(2)	(3)	(4)	(5)	(6)	(7)	(8)
		Pre-surgery	During surgery	Post-surgery	Disinfection	Packaging	Storage	Transport	Disposal
**≥0,3 μm**	**Mean ± SD**	1,156 ± 1,066	35,560 ± 60,399	3,493 ± 1,195	145,211 ± 99,112	193,302 ± 148,363	68,232 ± 89,042	41,390 ± 45,181	205,923 ± 162,187
	**Min—Max**	0–18,163	34–1,958,403	463–26,122	22,088–511,562	19,789–757,869	1,613–438,224	1,073–181,336	21,214–550,407
**≥0,5 μm**	**Mean ± SD**	662 ± 509	3,332 ± 4,775	1,578 ± 539	27,415 ± 39,012	65,697 ± 103,546	5,945 ± 7,362	3,955 ± 1,990	50,245 ± 74,451
	**Min—Max**	0–10,789	26–262,716	80–17,611	3,321–458,333	3,212–639,528	360–99,230	528–10,704	1,428–226,470
**≥1 μm**	**Mean ± SD**	495 ± 324	573 ± 363	1,196 ± 466	10,898 ± 15,771	35,036 ± 59,161	1,752 ± 2,056	1,501 ± 560	13,504 ± 19,817
	**Min—Max**	0–6,430	13–10,166	44–15,369	602–428,025	732–458,094	134–34,009	468–3,439	234–65,099
**≥5 μm**	**Mean ± SD**	46 ± 25	40 ± 23	114 ± 40	309 ± 337	746 ± 1,261	56 ± 10	87 ± 33	124 ± 78
	**Min—Max**	0–460	0–374	3–1,213	29–12,956	32–11,126	10–311	18–250	5–433
**≥10 μm**	**Mean ± SD**	40 ± 20	36 ± 23	93 ± 29	115 ± 86	140 ± 159	51 ± 12	94 ± 51	64 ± 39
	**Min—Max**	0–282	1–249	4–878	20–1,818	22–1,435	4–300	15–223	8–343
**≥25 μm**	**Mean ± SD**	9 ± 4	8 ± 4	20 ± 9	12 ± 8	12 ± 7	9 ± 2	17 ± 8	11 ± 7
	**Min—Max**	0–70	0–72	0–233	0–45	1–39	0–63	2–53	1–72

The mean values, standard deviations (SD), minimum (Min), and maximum (Max) values of airborne particle load with different diameters of all surgeries (average) were analyzed with a particle counter during use and reprocessing (1–8). Particle load is shown as numbers/m^3^ [no./m^3^].

## Discussion

The analysis during use and reprocessing of sterile goods in the present study demonstrated that properly reprocessed surgical instruments in sterile packaging systems (such as reusable containers) only play a minor role as a potential exogenous source of microbial contamination compared to the high microbial load in the air. To our knowledge, this is the first study, that analyzed the bacterial and particle load in the air, on surgical instruments, and on the outer surface of reusable containers through all steps of the use and reprocessing of sterile goods (from the OR to the CSSD).

### Instruments and reusable containers as a potential source of exogenous microbial contamination

In the present study, no microbial load (0 CFU) was detected on the working ends of reprocessed instruments analyzed within the OR (pre- (1), during (2), and post-surgery (3) (Table 1)), which is contrary to the literature, where microbial loads of 0–300 CFU were found during surgery [7–10]. Furthermore, it made no difference whether the analyzed instruments were used intraoperatively and were in contact with patient tissues, or whether they were left unused on the sterile table. Nevertheless, the presence of bacteria and fungi in the environment of these instruments within the OR was demonstrated by active and passive air sampling (Fig 2, Table 2). Different studies reported a correlation of the contamination between opened sterile operating-room trays and instruments to increased surgery time [13, 14, 21, 22]. However, Dalstrom et al. reported contamination of opened sterile operating-room trays after 30 minutes and described a direct correlation between the bacterial load and the time the trays were open [13]. Similarly, the contamination rate of unused but opened hemostats increased with time in a conventional OR compared to an OR with a laminar airflow system as reported by Ritter et al [14]. Furthermore, Benen et al. showed that unused instruments (crile clamps) outside of the direct laminar airflow had higher bacterial loads than surgical instruments within the direct laminar airflow [21].

The microbial load on surgical instruments was also analyzed within the CSSD to identify other sources of contamination of these instruments. After cleaning and disinfection (4) in a washer-disinfector, a maximum microbial load of 3 CFU was detected (Table 1). This is in contrast to higher values in the literature, which show substantially higher microbial loads (41–75 CFU/100 ml) [10]. However, a direct comparison of the microbial load of the instruments with literature data is limited as the studies were carried out in different countries, according to different standards, and with different sampling methods (such as contact agar plates, eluate of the whole instrument, or a swab test).

Furthermore, other studies demonstrated a relatively high incidence of microbial contamination after packaging and maintenance of reusable surgical instruments (25 CFU or 100 CFU/instrument) [11, 12], whereas the present study detected a maximal microbial load of 22 CFU (packaging (5)). The microbial load of surgical instruments was not analyzed directly after sterilization in the present study or in the literature. Alternatively, we analyzed the microbial load of sterile surgical instruments by opening sterile packaging systems (reusable containers with different storage times (1 week to 6 months)) in the sterile storage area. As there was no microbial load (0 CFU) at this step of reprocessing (storage (6)), it can be assumed that sterilization was efficient, even if some bacteria were still present on the instruments after cleaning and disinfection (4) or after packaging (5) with disinfected hands. Comparable literature data concerning this step of sampling is not available.

In the unclean area of the CSSD (disposal (8)), in contrast to the literature [10], a lower microbial and fungal load of 59 CFU could be analyzed on instruments. However, the surgical instruments in the present study did not come from only one instrument tray used in one surgery. Therefore, it is possible that some instruments within the unclean area of the CSSD (disposal (8)) were contaminated during other surgeries, such as gynecological or gastro-intestinal surgery. A future study could investigate a specific instrument tray over a complete use and reprocessing cycle. The most common contaminating microorganism was coagulase-negative staphylococci, followed by *Bacillus* species and *Micrococcus* species, which was in accordance with the literature [9–12].

Since reusable surgical instruments are sterilized and stored in sterile packaging systems (such as reusable containers) until they are used in the operating theatre, the microbial load on the outer surface of the reusable containers of three different manufacturers with different ages and different conditions was analyzed too. Data of this kind hast not been measured or published before. The present study`s data summarized in Table 1, shows a high microbial load on the outer surface of reusable containers at every step of the use and reprocessing. However, no microbial load (0 CFU) could be detected on the sterile instruments, which were analyzed from opened reusable containers in the sterile storage area (storage (6)) and immediately before surgery (pre-surgery (1)). This indicates that the reusable containers function as a sterile packaging system and prevent microbial contamination of the sterile instruments they contain.

### Air as a potential source of exogenous microbial contamination

The air still seems to be the primary exogenous source of microbes that could potentially settle on properly reprocessed surgical instruments in this study. However, we detected a low microbial load on the used settle plates (Table 2), which is similar to already published data (during active surgery: 12–17 CFU [21–23]). The limitations of using settle plates for passive air sampling are well known [24]. Literature concerning the other stages of the sterile-goods-cycle are to the best of our knowledge not known.

The analysis of the active air sampler showed a high microbial and fungal load across every step of use and reprocessing (Fig 2), which was comparable to previous data. However, in the literature, the results varied and depended on the type of surgery, the country where the surgery was performed, and which active air sampler and flow rate was used. Even ahead of the first surgery of the day (before people entered the operating room), the microbial load was not near zero. The present study detected an average microbial load of 39 CFU/m^3^ (Fig 2, pre-surgery (1)), similar to the literature data (38 CFU/m^3^) [25]. During surgery (2), literature data differs between 25–287 CFU/m^3^ [11, 21, 26–28], whereas the present study showed an average microbial load of 27 CFU/m^3^. The highest average microbial load was measured after surgery (post-surgery (3)) with 56 CFU/m^3^. The average microbial load within the CSSD was even lower (38 CFU/m^3^ directly after cleaning and disinfection (4) and 52 CFU/m^3^ on packaging tables (5)). This data is similar to literature data [29, 30]. Only one other study analyzed the air within the sterile storage area (storage (6)). It showed a microbial load of 18–230 CFU/m^3^ [31], which contradicts the results of the present study, which detected an average microbial load of 32 CFU/m^3^. Further, published data analyzing the airborne microbial load on intra-hospital transport ways (7) and in the unclean area of the CSSD (disposal (8)) are not available. Our study showed a maximum microbial load of 52 CFU/m^3^ and 50 CFU/m^3^ in these areas respectively.

The 107 air samples taken in this study also identified coagulase-negative staphylococci (n = 1,223; 28%) as the most common pathogen. Additionally, some non-pathogenic fungi were cultured from the air samples. The bacterial pattern of the most commonly identified bacteria (including non-pathogenic bacteria), such as Micrococcus species or Bacillus species, was largely consistent with that found in the literature [23, 28, 31].

The particle load was also analyzed during the use and reprocessing of the sterile goods (Table 3) as bacteria are linked to organic and inorganic particles [15–17]. Correlating to the highest microbial load in the air (Fig 2), the highest average particle load of ≥0.3 μm particles was detected during surgery (2) 1,958,403 no./m^3^ within the OR. However, other studies analyzed different particle loads during surgery with different sizes, such as ≥0.5 μm (4,194,569 no./m^3^) and ≥5 μm (46,262 no./m3–13,519 no./m^3^) [26, 27]. It was also described that the particle load in an empty operating theatre is analogous to the microbial load, to close to zero [25]. The present study detected a maximum particle load of 10,789 no./m^3^ for particles of ≥0.5 μm diameter pre-surgery (1). Further literature data regarding the other steps of reprocessing are, to the best of our knowledge, not available. A direct comparison of this data is difficult because different particle samplers with different flow rates and light scattering methods were used to measure different particle sizes. Nevertheless, the high microbial and particle loads detected in the air of the OR (1–3) are consistent with findings in the literature. The air remains an important exogenous source of microorganisms potentially leading to surgical site infections. Properly reprocessed reusable surgical instruments and sterile packaging systems did not show any signs of contamination in this study.

### Further exogenous sources of microbial contamination

It was previously demonstrated that most SSIs are causally related to the time spent in the operating room rather than in postoperative care [32]. A direct correlation between the air and postoperative infections and the importance of a suitable OR ventilation system to reduce the microbial load was described in different studies [18–20, 33, 34]. For example, a reduction of the microbial count in the wound area from 23.5 CFU/m^3^ without laminar airflow to 3.5 CFU/m^3^ with a mobile laminar airflow was reported by Sossai et al [35]. However, recent studies discuss whether the laminar airflow system significantly impacts infection rates [36–40].

Furthermore, it is commonly known that the microbial load of the operating theatres depends on the number of people inside the OR [41–45]. Within the present study (Fig 2) and the study performed by Landrin et al. [25], confirmed this as a low microbial load was measured at rest (pre-surgery (1)), which increased when people entered the OR. Further studies demonstrate a direct positive correlation between the traffic flow (opening and closing the doors) and the number of persons present in the OR. These factors positively correlate with the microbial load in the air [45–47]. Additionally, it could be demonstrated that an increased duration of surgery, e.g., greater than three hours, was associated with a 7.5-fold increased risk of SSI [39]. That bacteria are located on the normal human skin flora, especially on the opening of hair follicles and comedones, was described by Montes and Wilborn [48]. Swab test analysis of the foreheads, eyebrows, and ears of operating theatre staff, whose facial area was not covered with theatre clothing, demonstrate the highest microbial count from the ear samples. Primarily, *Staphylococcus aureus* and coagulase-negative staphylococci could be identified [49]. One study even described a hospital-acquired outbreak of methicillin-resistant *Staphylococcus aureus* (MRSA) infection spread by a surgeon carrier [50]. Theatre staff could also carry other pathogenic bacteria like every other person, such as methicillin-resistant *Staphylococcus epidermidis* (MRSE) [51]. This demonstrates the importance of wearing the correct clothing within the OR. However, the type of clothing within the OR is controversially discussed [42, 51]. Face masks are a standard method of preventing the respiratory transmission of bacteria by breathing. Charnley recommended the usage of sterile helmets with masks and body exhaust suits to reduce the microbial load within the OR. Another study demonstrated comparable microbial loads when disposable head, mask and helmet aspiration systems were used, but increased microbial loads were measured when headgear was omitted entirely [41]. Whether surgical face masks significantly influence the microbial load in the air is also discussed controversially [43, 47].

Another potential source of SSI-causing bacteria is the surgeon´s hand and the operating theatre staff`s hands. Therefore, hand disinfection and gloves are utilized to prevent the transmission of bacteria from operating theatre staff to patients. In orthopedic surgery, for example, double gloving is recommended in case gloves are perforated [52]. This data emphasizes the importance of hand hygiene and surgical hand preparation in the OR. Different guidelines, strategies and products for surgical hand preparation are described [53–55], however the use of an alcohol-based hand disinfectant is the standard for effective hand hygiene in hospitals [53, 56].

Even if the present study did not analyze the microbial load of disinfected hands and skin of the theater staff, it showed that properly reprocessed surgical instruments and sterile packaging systems were not the main source of microbial contamination, as no microbial load was detected on them.

### Limitations of the study

Our study has several limitations. The applied convenient sample method considerably limits the generalizability of the findings. Furthermore, the small sample size could have provoked a type 2 error, i.e., small and medium effects might not be detected due to the inadequate sample power. The number of airborne microbial load measurements during surgery (2) differed due to the different duration of the surgeries. Hence, we compared the first two measured values during surgery (15 and 30 minutes into the surgery) with those measured during other steps, as a minimum of two measurements were performed during each surgery. Furthermore, the number of measurements after the different surgeries (post-surgery (3)) also varied. We used the first measurement for the longitudinal analyses respectively, as at least one measurement was performed in this step. Therefore, the generalization and causal interpretation of the changes in microbial load between the steps pre-, during, and after surgery (1–3) is limited since we could not consider all the measurements performed.

Furthermore, the interpretation of the descriptive and longitudinal analyses regarding microbial load on the instruments across the different use and reprocessing steps is also limited since the measurements were not performed on the same instruments. It is possible that some instruments were already contaminated in the previous steps or during other surgeries, but they were not sampled. The related-sample tests regarding the differences in microbial load on the outer surface of reusable containers and on instruments taken from those containers were used because the measurements refer to the same pair (on the outer surface of reusable containers vs. instruments). As the measurements took place during the same step of use and reprocessing, the causal interpretation of the findings is not eligible due to the cross-sectional design of the analyses. Since only one measurement was taken on the outer surface of each reusable container, the number of measurements varied. We used the mean values of the measurements on the instruments. Therefore, the measurements were compared with different reliability levels. Despite these limitations, the present study, the nature of which is exploratory, provides first insights into the microbial load in the air, on instruments, and the outer surface of reusable containers throughout use and reprocessing.

## Conclusion

This study demonstrates that air is a greater source of microbes (such as bacteria and fungi) compared to properly reprocessed reusable surgical instruments and sterile packaging systems, which can potentially lead to SSIs. However, data on bacterial pathogenicity and relevant infection rates in patients after surgery is lacking.

## Supporting information

S1 File Raw data of microbial load analyzed on instruments, on the outer surface of sterile containers, on the outer surface of sterile containers and on instruments taken from the same sterile container, in the air, and on settle plates. In sheet 1 the microbial load (CFU) analyzed on surgical instruments from three hospitals on different sampling days is listed. Sheet 2 lists the microbial load (CFU) detected on the outer surface of sterile containers. In sheet 3 the microbial load (CFU) measured in the sterile storage area (sampling step: storage 6) on the outer surface of sterile containers and on instruments taken from the same container is listed. Sheet 4 lists the airborne microbial load (CFU/m^3^) detected via active air sampling. In sheet 5 the microbial load analyzed on settle plates (by passive air sampling) is listed.
(XLSX)

S2 File Raw data of airborne particle load measured via particle counter. Within the different sheets the airborne particle load (no./m^3^) analyzed on different sampling days within different clinics are listed.
(XLSX)
